# The association of FKBP5 polymorphisms with the severity of depressive disorder in patients with methamphetamine use disorders

**DOI:** 10.3389/fpsyt.2023.1147060

**Published:** 2023-03-27

**Authors:** Ting Fang, Meng-Nan Liu, Xiao-Yu Tian, Guan-Yi Lu, Fei Li, Xiaojie Zhang, Feng Liu, Wei Hao, Ning Wu, Hong Li, Jin Li

**Affiliations:** ^1^State Key Laboratory of Toxicology and Medical Countermeasures, Beijing Key Laboratory of Neuropsychopharmacology, Beijing Institute of Pharmacology and Toxicology, Beijing, China; ^2^Medical School of Chinese PLA, Beijing, China; ^3^Department of Psychiatry, National Clinical Research Center for Mental Disorders, The Second Xiangya Hospital of Central South University, Changsha, Hunan, China; ^4^Compulsory Detoxification Center of Changsha Public Security Bureau, Changsha, Hunan, China

**Keywords:** depressive disorder, FKBP5, linkage disequilibrium, methamphetamine use disorder, single nucleotide polymorphism

## Abstract

**Background:**

Co-occurring depressive disorder (DD) in patients of methamphetamine use disorder (MAUD) impacts the diagnosis, treatment, and prognosis of the disease. Although FKBP5 has been associated with a variety of psychiatric disorders, whether FKBP5 influences depression susceptibility in MAUD is unknown so far.

**Methods:**

Here, we sequenced six FKBP5 single-nucleotide polymorphism (SNP) sites (rs4713916, rs6926133, rs9470080, rs737054, rs4713902, and rs9470079) in 282 methamphetamine users. MAUD and DD were evaluated by clinical questionnaires. SPSS was used to analyze the relationship between FKBP5 SNPs and DD in individuals with MAUD.

**Results:**

Of the 282 methamphetamine users, 161 individuals met the MAUD criteria, and among them, 50 patients (31.1%) had DD co-occurring. Importantly, the incidence of DD in individuals with MAUD was 3.314 times greater than that of the methamphetamine users who did not meet the MAUD criteria (*p* < 0.001). Although none of the six SNPs of FKBP5 were correlated with the co-occurrence of DD in the population with MAUD, two FKBP5 alleles (rs4713916A and rs6926133A) were substantially associated with the higher DD scores in patients with MAUD (*p* < 0.05). Moreover, those with the two risk alleles do not have much higher scores than those with a single risk allele, and the strong linkage disequilibrium of the two SNPs may be the underlying cause of this result. Despite having weak linkage disequilibrium with either rs4713916 or rs6926133, FKBP5 rs9470079 became risky when paired with either.

**Conclusion:**

The results of this study revealed that the FKBP5 risk alleles (rs4713916A and rs6926133A) were associated with a greater probability of severe DD in patients with MAUD. These findings here would help with the development of biological early warning markers and the creation of personalized treatment strategies for MAUD.

## Introduction

Methamphetamine (MA) developed as an amphetamine derivative, is a potent psychostimulant substance with a significant potential for addiction. Overall, 61% of MA users relapse to MA abuse within a year after finishing treatment programs. Furthermore, only 13% of people maintained abstinence from MA for 5 years. MA produces a strong central stimulatory effect, which will lead to MA use disorder (MAUD) (1, 2). The long-term use of MA frequently leads to serious central nervous system impairment, which in turn causes mental disorders, auditory hallucinations, paranoia, delusions, and other symptoms (3, 4), and these symptoms may result in serious criminal offenses such as troublemaking, robbery, assault, and murder. It may also cause the spread of infectious illnesses such as hepatitis and AIDS (5–7). MAUD, therefore, poses a substantial threat to public health, social stability, and national security, in addition to causing severe physical and emotional harm to individuals (8).

Research shows that dual diagnosis of depression and MAUD worsens the overall prognosis and requires a special treatment approach (9). Depression and substance use disorders frequently coexist. According to a study conducted in the USA, people who use drugs or alcohol are nine times more likely to suffer from serious depression than people who do not (10). Similar studies showed that those with alcohol or drug use disorders were three to four times more likely to have experienced depression than the general population (11, 12). On the other hand, those who have depression also have a higher tendency to use psychoactive substances. Nearly one-third of patients with major depressive disorders also have co-occurring drug use disorders, which increases the risk of suicide and the severity of their mental problems (13, 14).

There is strong evidence that depression participates in the pathophysiology of psychostimulant addiction. The hypothalamus–pituitary–adrenal (HPA) axis is crucial to the development of both disorders (15). The HPA axis is stimulated by MA (16, 17). Plasma cortisol concentrations were shown to be elevated in a human investigation following acute MA exposure using an intravenous dosage of 0.5 mg/kg MA (18). Chronic MA abuse can also alter HPA axis function. Methamphetamine users had significantly lower basal plasma cortisol concentrations, but similar basal plasma oxytocin and arginine vasopressin concentrations compared with controls (19). These changes in the HPA axis function are linked to altered stress-related behaviors and may have an impact on the development of addictive behaviors. In addition, studies have shown that serious depression causes hyperactivity in the HPA axis (20–23). HPA axis genetic variation and activity were important predictors of cognition across the entire sample of depressed subjects and healthy controls (20).

The HPA axis consists of stimulating forward and feedback inhibition loops involving the hypothalamus, pituitary, and adrenal glands to produce glucocorticoid end products (15). FKBP5 is a 51-kDa immunophilin that belongs to the family of FK506-binding proteins. FKBP5 consists of an FKBP-type peptidyl-prolyl cis-trans isomerase (PPIase) domain (called FK1), an FKBP-like domain (FK2), and a three-unit repeat of the tetratricopeptide repeat (TPR) domain, which may bind the MEEDV motif of other proteins (24, 25). FKBP5 is a stress response protein that regulates HPA axis function in stress reactivity. Single-nucleotide polymorphism (SNP) of the FKBP5 gene can change the expression of the FKBP5 protein (26, 27). Overexpression of FKBP5 decreases hormone binding affinity and nuclear translocation of glucocorticoid receptors (GRs), thereby reducing GR sensitivity (28–30). After exposure to trauma, reducing GR sensitivity will impair HPA axis negative feedback, which will lead to an increase in cortisol levels. Cortisol is linked to a variety of stress-related mental disorders, including psychosis (31), borderline personality disorder (32), anxiety (33, 34), schizophrenia (35), depression (36, 37), PTSD, and suicidal tendencies (38, 39).

Given that both MAUD and depression are associated with HPA axis dysregulation, does FKBP5 SNP affect depression susceptibility in individuals with MAUD? To answer this question, FKBP5 SNPs were sequenced in 282 methamphetamine users. In this investigation, the association between the six FKBP5 SNPs and the severity of depression was examined.

## Materials and methods

### Ethics statement

This study was conducted among MA users. The Second Xiangya Hospital Ethics Committee of Central South University gave its approval for investigation (No. 2017-064). After the individuals had received a thorough explanation of the study, signed informed consent was acquired.

### Participants of MA users

Participants were recruited from a compulsory drug rehabilitation facility in Changsha, China, between December 2017 and September 2019. All participants were Han Chinese. The inclusion criteria for the MA users were: (1) age ranging from 18 to 60 years, (2) at least 14 days of detoxification at the time of study participation, and 3) MA was the main illicit substance that was abused. Participants who had serious mental illness or considerable cognitive impairment before using MA were excluded from the research. During that period, a total of 326 individuals were enrolled in this study, including 300 individuals with MA as the primary abused substance, but only 282 individuals agreed to donate their blood samples. Consequently, the sample size of this study was 282 individuals.

### Assessment of MAUD and DD

Each subject among the 282 MA users underwent a semi-structured interview with two skilled and experienced psychiatrists. The diagnosis was based on the Semi-structured Assessment for Drug Dependence and Alcoholism (SSADDA, Chinese version). Our group translated the English version of the SSADDA, which was developed based on DSM-5 criteria into Chinese and evaluated its interrater reliability and concurrent validity in patients with MAUD, and the result showed that the Chinese version of the SSADDA has good reliability and validity among Chinese MA users (40). The MAUD part included 11 items (drug use time, health condition, quit experience, intent, tolerance, withdrawal, activities, craving, hazard, neglect, and social). Each item received a score of 1, so its score scales ranged from 0 to 11. MAUD was identified in those who received scores of 2 or above (40). Similarly, a skilled clinician working with SSADDA evaluated the participants' depression rating scales, which consisted of nine items based on DSM-IV criteria. These items covered the clinical symptoms of depression, including depressive mood, loss of interest, changed appetite (poor appetite or overeating), sleep disturbance (insomnia or sleeping too much), psychomotor agitation or retardation, fatigue or loss of energy, feelings of worthlessness or excessive or inappropriate guilt, diminished ability to think or concentrate or indecisiveness, and thoughts of death, so depression scores range from 0 to 9. Those who had a score of 2 or greater were considered to have DD (41), and a higher score represented more symptoms and greater severity of DD. The demographic information for the 282 participants is described in Table 1.

**Table 1:** Description of the analyzed population.

**Variables**	**Non-DD (*n =* 221)**	**DD (*n =* 61)**
**Demographics**		
Sex (% male)	198 (89.6)	59 (96.7)
Age (years), mean ± SD	35.33 ± 6.71	34.56 ± 5.85
High school or above (%)	53 (24.0)	12 (19.7)
Married (%)	158 (71.5)	41 (67.2)
Full-time job (%)	143 (66.2)	42 (68.9)
**Depression characteristics**		
Age of onset (years), mean±SD	-	30.95 ± 6.37
Duration of depressive episodes (weeks), mean ± SD	-	6.05 ± 11.28
Cooccurring MAUD (%)	111(50.2)	50 (82.0)
Cooccurring AUD (%)	131 (59.3)	30 (49.2)
Cooccurring TUD (%)	211 (95.5)	60 (98.4)

Demographic and clinical features between methamphetamine (MA) users without and with depressive disorder (DD).

### Blood sample collection and DNA extraction

Each participant had 10 ml of blood drawn into an ethylenediaminetetraacetic acid anticoagulant tube, which was then quickly centrifuged at high speed to separate the blood cells. DNA was isolated from blood cells using the Genome Extraction Kit (Wuhan NanoMagBio Technology Co., Ltd.). In brief, the blood sample was first broken down with a lysate and protease K. After the lysis was completed, the liquid was transferred into a centrifuge tube containing magnetic beads, isopropyl alcohol was added, and then the content was mixed by vortexing. At this point, the sample was combined with magnetic beads, and the magnetic beads were attracted by magnetic racks during washing. After drying to complete the evaporation of ethanol, the eluent was added to remove the nucleic acid from the magnetic beads, and the DNA sample in the liquid was obtained.

### Genotyping

The six FKBP5 SNPs (rs737054, rs6926133, rs4713902, rs9470080, rs9470079, and rs4713916) selected in our study span 94.5 kb across FKBP5 locus. Genotyping was performed using the Applied Biosystems 7900HT TaqMan genotyping platform (Applied Biosystems, Foster City, Calif). Supplementary Table 1 lists the forward and reverse primer pairs that were utilized for the experiment. The following steps were used in the RT-qPCR procedure: 5 min at 96°C; 10 cycles of 20 s at 96°C, 30 s at 62°C (−1°C/cycle); 30 s at 72°C; 35 cycles of 20 s at 96°C, 30 s at 52°C; 30 s at 72°C; 10 min at 72°C; and finalization at 4°C, indefinitely.

### Statistical analyses

Data analysis was performed using SPSS 25 (IBM, Armonk, NY) and GraphPad Prism (GraphPad Software, Inc., La Jolla, CA). Two-tailed Pearson's chi-square (χ^2^) test was employed to test variations in the genotypic distribution between the groups of DD cases and control participants. The Mann–Whitney *U*-test was used to test the difference in the depression scores between two groups of different genotypes. The linkage disequilibrium (LD) structure of the SNPs was determined by the Genetics and LD heatmap Packages in R version 4.0.0.

## Results

### Characteristics of participants

In our study, 282 participants of MA users were recruited. Their clinical characteristics and demographics are summarized in Table 1. Among these, 61 patients (21.6%) were diagnosed with DD. The average age of the patients with DD was 34.56 ± 5.85 years, while the average age of the patients without DD was 35.33 ± 6.71 years. Twelve patients with DD (19.7%) had completed high school education, 41 subjects (67.2%) were married, and 42 subjects (68.9%) had full-time jobs. Their average age at depression onset was 30.95 ± 6.37 years, and the average duration of depressive episodes was 6.05 ± 11.28 weeks. The proportion of MAUD morbidity was 82.0% among the 61 patients with DD and 50.2% among patients without DD. The comorbidities of alcohol use disorder and tobacco use disorder in the 61 patients with DD were 49.2 and 98.4%, respectively.

**Figure 1:**
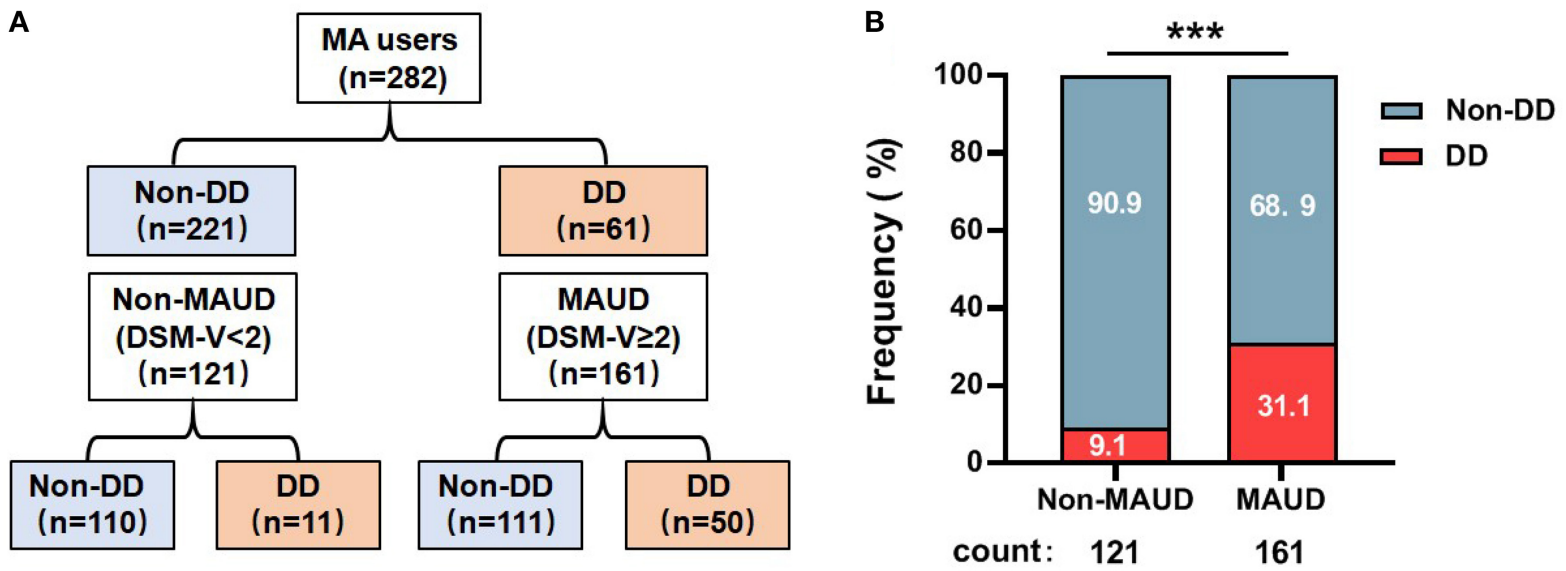
Frequency of individuals with DD among the patients with MAUD. **(A)** The structure of the 282 MA users involved in our research. A total of 161 individuals were diagnosed with MAUD; among them, 50 individuals had DD. **(B)** The results of the proportion of DD individuals among patients with MAUD (DSM-V≥2) and MA users only (DSM-V< 2), ***p < 0.001; chi-square test. MA, methamphetamine; MAUD, methamphetamine use disorder; DD, depressive disorder.

A total of 161 out of 282 methamphetamine users were patients with MAUD, and 50 of them also had co-occurring DD (Figure 1A). To our interest, the prevalence of DD morbidity was 3.314 times greater in patients with MAUD than in those without MAUD (31.1 vs. 9.1%, *p* < 0.0001) (Figure 1B). This result suggested that the patients with MAUD were vulnerable to suffering from DD.

### Genotype distributions of participants with MAUD

**Figure 2:**
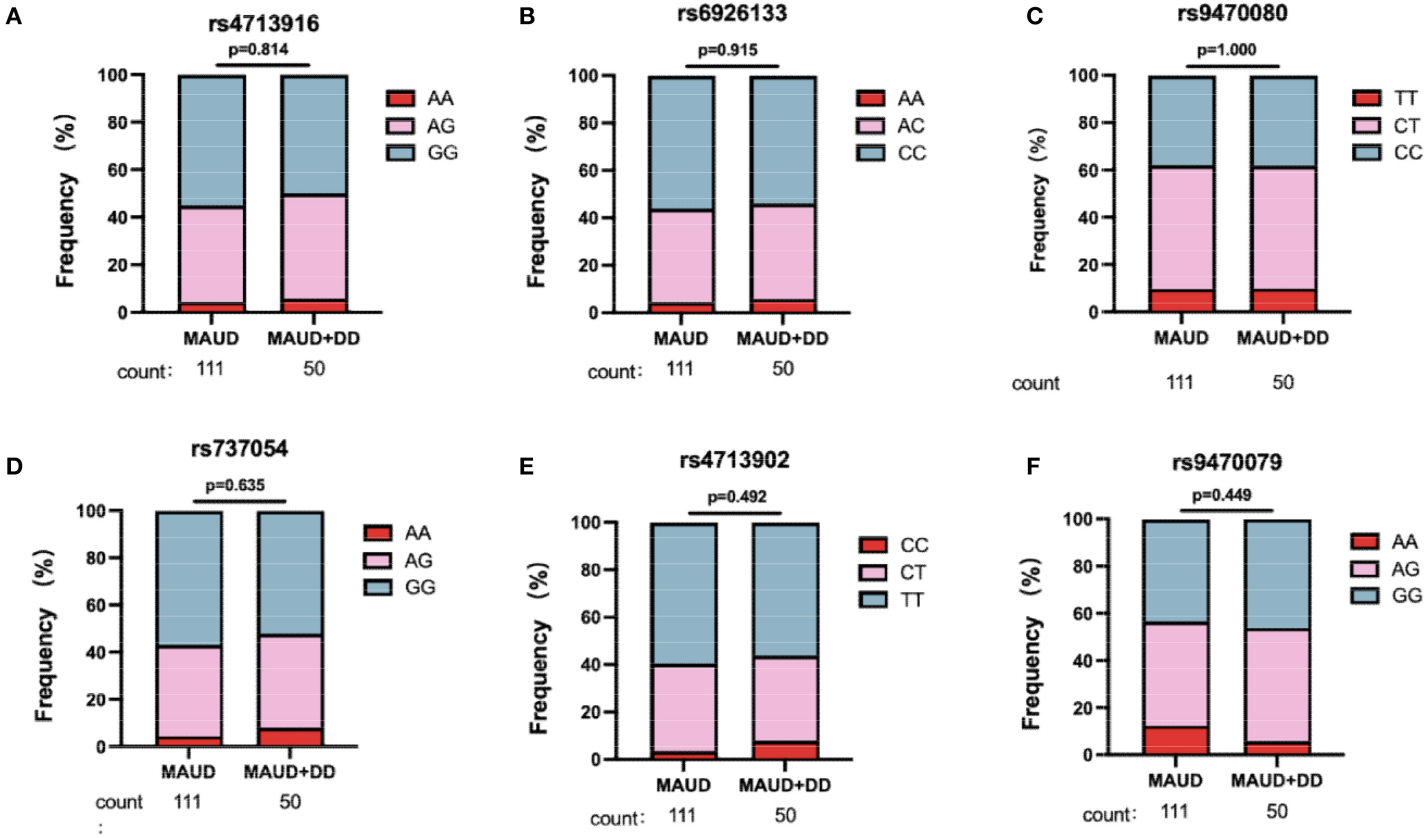
Genotypic distribution for six FKBP5 SNPs. In 161 patients with MAUD, 50 individuals were comorbid with DD. Genotypic distribution of FKBP5 SNP rs4713916 **(A)**, rs6926133 **(B)**, rs9470080 **(C)**, rs737054 **(D)**, rs4713902 **(E)**, and rs9470079 **(F)** in 111 MAUD patients without DD and 50 MAUD patients with DD. There was no significant difference in the distribution of the six SNP alleles between the two groups, which hinted that the six FKBP5 SNPs were not associated with the occurrence of DD in patients with MAUD (chi-square test). MAUD, methamphetamine use disorder; DD, depressive disorder.

Six FKBP5 SNPs (rs737054, rs6926133, rs4713902, rs9470080, rs9470079, and rs4713916) were genotyped in the 282 participants of MA users. Those SNPs have been previously implicated in schizophrenia (41), responses to stress (42), and depression symptoms in bipolar disorder (43). All six SNPs were in Hardy–Weinberg equilibrium (HWE) (*p* > 0.05) (Supplementary Table 2), which indicated that the SNPs were suitable for studying the genetic association of diseases (44). Figure 2 illustrates the contribution statuses of FKBP5 SNPs between MAUD patients with or without DD. For each of the six FKBP5 SNPs, there were no appreciable variations in genotype distribution. According to these findings, none of the six FKBP5 SNPs significantly affected the occurrence of DD in patients with MAUD.

### Association of FKBP5 SNPs with the depression severity in patients with MAUD

**Figure 3:**
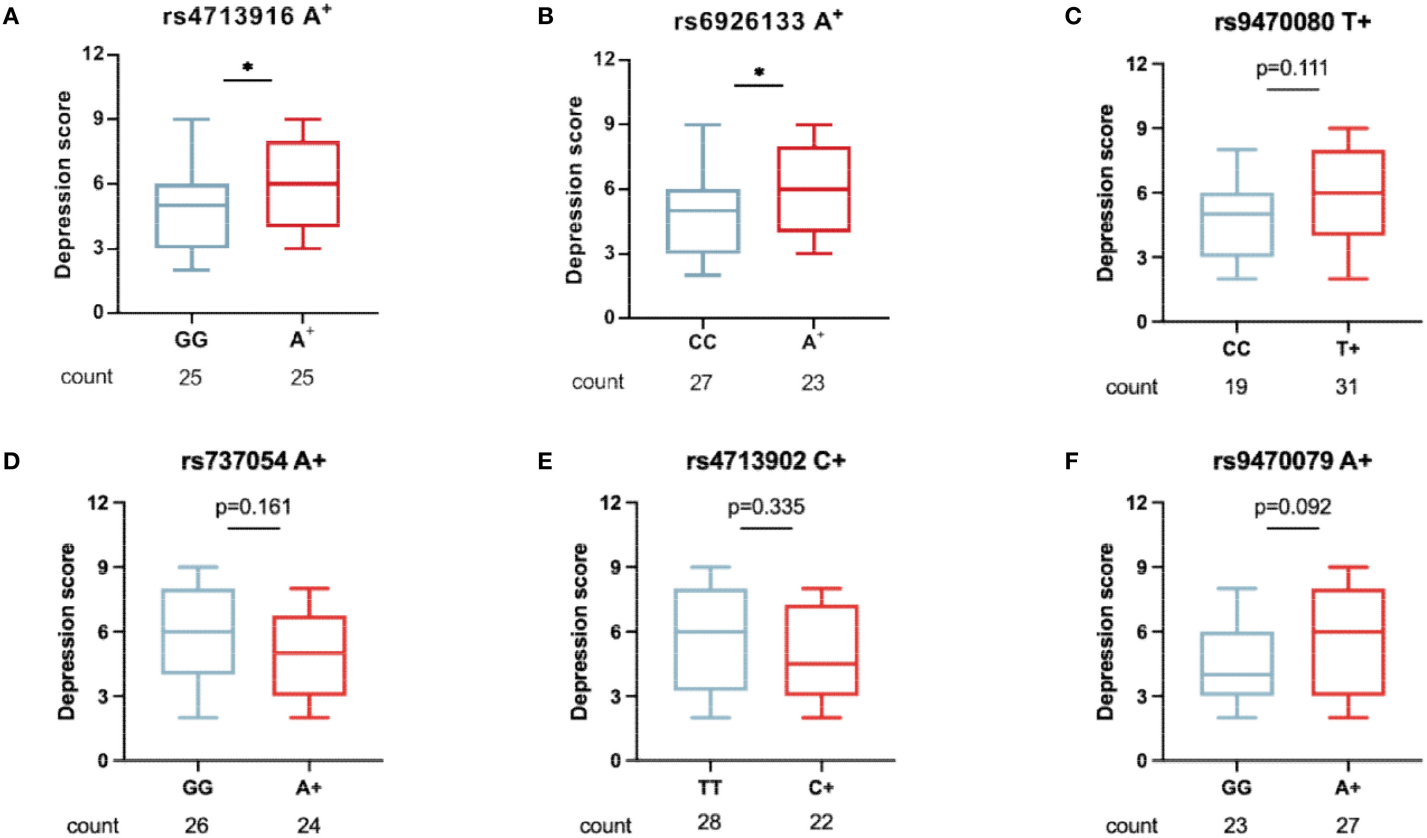
The association of the six FKBP5 SNPs with DD severity in patients with MAUD. In 50 patients with DD who had comorbid MAUD, the association of the minor alleles of FKBP5 rs4713916 **(A)**, rs6926133 **(B)**, rs9470080 **(C)**, rs737054 **(D)**, rs4713902 **(E)**, and rs9470079 **(F)** with the depression scores was measured. Patients carrying FKBP5 rs4713916A or rs6926133A had greater depression scores. *p < 0.05, n.s., no significance. Mann–Whitney *U*-test. MAUD, methamphetamine use disorder; DD, depressive disorder.

**Figure 4:**
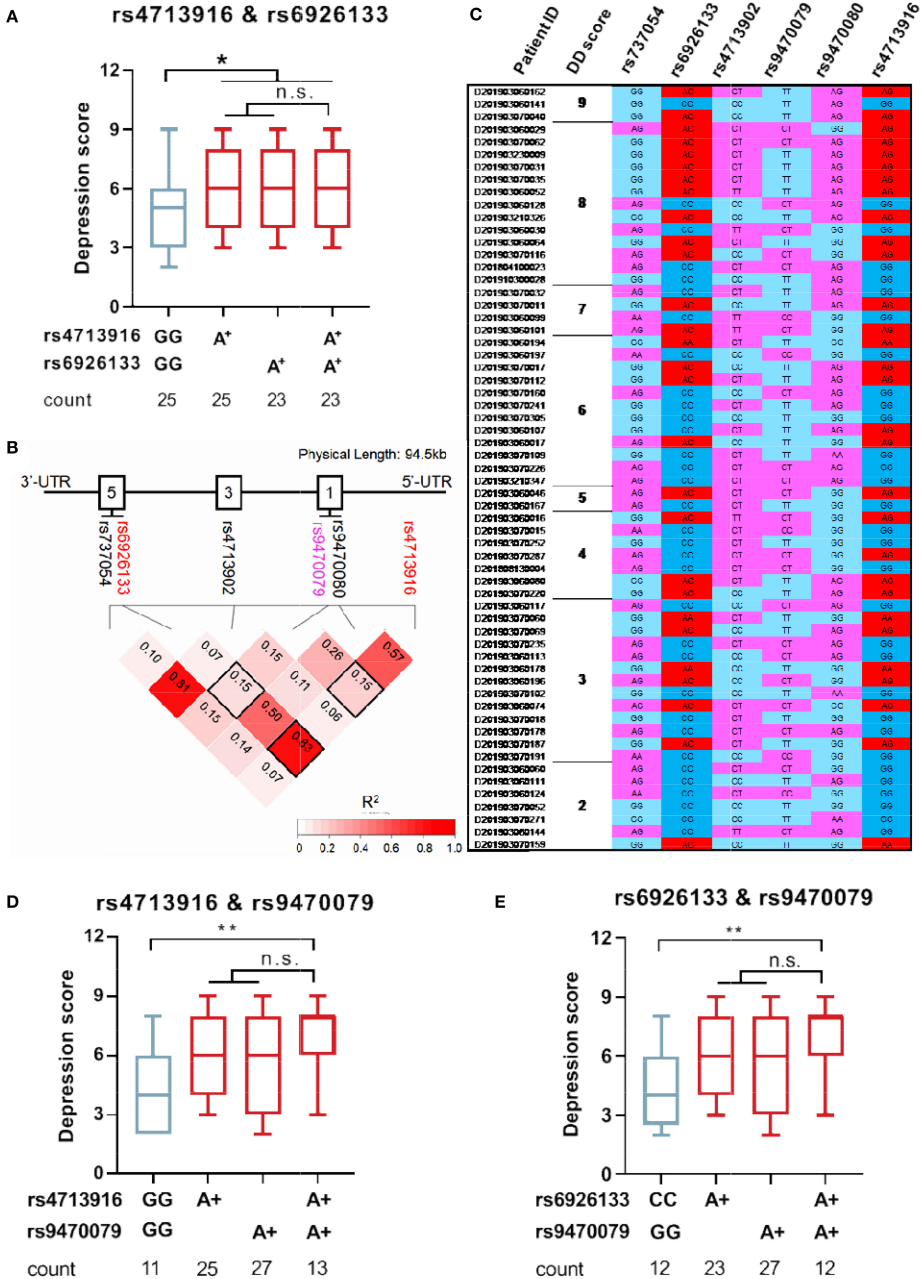
FKBP5 risk alleles rs4713916A and rs6926133A can serve as biomarkers of severe DD in patients with MAUD. **(A)** Depression status in the individuals carrying FKBP5 rs4713916 A and/or rs6926133A risk alleles. *p < 0.05; n.s., no significance; Kruskal–Wallis test. **(B)** The chromosomal location of the six FKBP5 SNPs on chromosome 6 is shown by the X-axis. The location distance is 94.5 kb. The intron structure of the gene is depicted as empty boxes on the top. The numbers in the pairwise LD block squares are R^2^ values. Complete LD (R^2^ = 1) and no LD (R^2^ = 0) are shown in red and white, respectively. Analysis was performed on 282 people. FKBP5 rs4713916 and rs6926133 had R^2^ values of 0.83 and were in strong LD. **(C)** The exhibition of DD scores and SNP information in 50 patients with MAUD. MAUD, methamphetamine use disorder; DD, depressive disorder; LD, linkage disequilibrium. **(D)** Depression status in the individuals carrying FKBP5 rs4713916A and/or rs9470079A risk alleles. *p < 0.05; n.s., no significance; Kruskal-Wallis test. **(E)** Depression status in the individuals carrying FKBP5 rs6926133A and/or rs9470079A risk alleles. *p < 0.05; n.s., no significance; Kruskal-Wallis test.

To understand whether FKBP5 SNPs were associated with depression severity in patients with MAUD, the comparison of depression scores between the two groups (patients carrying minor alleles vs. those carrying no minor alleles) was conducted. Patients carrying FKBP5 rs4713916A or rs6926133A had greater depression scores (*Z* = –2.003, *p* = 0.0459 and *Z* = –2.108, *p* = 0.0353, respectively), suggesting that those SNPs may be risk alleles for the development of severe DD (Figures 3A, B). The depression scores of the groups between the individuals holding zero, single, or two risk alleles were assessed to ascertain whether those carrying two risk alleles have more severe DD. Although those with the rs4713916A and rs6926133A haplotypes (A-A group) had higher scores than those without risk alleles (G-G group) (*Z* = –2.088, *p* = 0.036), the individuals of the A-A group did not exhibit significantly greater depression scores than those with the heterotype of rs4713916A or rs6926133A (Figure 4A). Furthermore, the six SNPs covered 94.5 Kb of the FKBP5 gene. The R^2^ values of the linkage disequilibrium (LD) ranged from 0.07 to 0.83. FKBP5 SNPs rs4713916 and rs6926133 were in a block of the strongest LD (R^2^ = 0.83). Within the pairwise block, the two dominant haplotypes, AA and GG (rs4713916 and rs6926133), accounted for 96% of the haplotype diversity (Figure 4B). Information on depressive scores and the six SNPs in the 61 patients with DD is shown in Figure 4C. More interestingly, even though FKBP5 rs9470079 had weak LD with either rs4713916 or rs6926133 (Figure 4B), it became risky when paired with either rs4713916 or rs6926133 (Figures 4D, E).

## Discussion

FKBP5 is a well-known modulator of the negative feedback loop of the HPA axis and has been linked to a variety of stress-related psychiatric diseases. However, little is known about whether it affects the development of DD in patients with MAUD. Our study here demonstrated that the FKBP5 SNPs, rs4713916A and rs6926133A, were associated with severe DD susceptibility in patients with MAUD.

FKBP5-induced GR insensitivity results in hypercortisolism due to a compromised feedback inhibition, which should be most pronounced when the HPA axis is activated (45, 46). Accumulating studies have demonstrated that FKBP5 SNPs are strongly related to several psychological affective diseases, including posttraumatic stress disorder (PTSD), borderline personality disorder (32), and suicidal tendencies (38, 39). According to Ising et al. (47) those who are homozygous for certain FKBP5 minor alleles, such as rs4713916, rs1360780, and rs3800737, have decreased cortisol recovery and higher levels of self-reported anxiety after psychosocial stress (47). Four SNPs of the FKBP5 gene in 3890 US service members deployed to Iraq and Afghanistan were examined. The results showed that probable PTSD subjects were significantly more likely to carry the alleles of rs3800373A, rs9296158G, rs1360780C, and rs9470080C. In addition, the A-G-C-C haplotype was significantly higher in probable PTSD subjects than in non-PTSD subjects (*p* < 0.05) (48). Similar findings were found in a study of 1,140 persons who lived through the 2008 Wenchuan earthquake in China, which showed that the rs3800373-rs9296158-rs1360780-rs9470080 haplotype A-G-C-T was substantially related to combined PTSD and depressive symptoms (37). Five FKBP5 SNPs (rs3800373, rs9296158, rs737054, rs1360780, and rs9470080) were genotyped in a sample of 101 unrelated Caucasian patients with BPD and 111 ethnically matched healthy controls. The findings indicated that borderline personality disorder was significantly correlated with each of the five FKBP5 polymorphisms (49).

FKBP5 SNPs are highly correlated with depression development and responsiveness to antidepressant drug treatment. Previously, a meta-analysis of 16 independent studies including 5,125 patients with depression and 8,399 controls showed that the FKBP5 rs1360780 and rs4713916 polymorphisms were associated with depression, but there was no significant association for FKBP5 rs9470080 or rs9296158 (36). FKBP5 rs1360780 T^+^ was significantly correlated with major depression, according to another meta-analysis finding (50). Moreover, there was a pronounced reduction in FKBP5 gene and FKBP51 protein expression in patients with depression after 4 weeks of antidepressant treatment, but there were increases in nonresponders. Only the FKBP5 rs1360780 T^+^ patients experienced a statistically significant treatment effect, indicating a superior response to antidepressant medication treatment. Thus, FKBP51 may be a suitable target for the development of antidepressant drugs (51). In our study, there was no significant difference in the distribution of the six SNP genes between the two groups, which hinted that the six FKBP5 SNPs were not associated with the occurrence of DD in patients with MAUD, and the sample size should be enlarged in future investigations.

Our study focused on the relationship between FKBP5 SNPs and depression comorbidity in patients with MAUD (Figure 1), which commonly co-occurs and has a bad prognosis (52, 53). The two disorder states interact in both directions. Substance use can result in negative mood changes and act as a compensatory behavior to lessen depression symptoms, thus indicating a process with worse outcomes than either disorder alone (54). Despite the information confirming the prevalence of depression among MA users, little is known about the available treatments. Therefore, the development of biomarkers for the presence of depression in MAUD is necessary (55). In 282 MA users, we sequenced six SNPs (FKBP5 rs4713916, rs6926133, rs4713902, rs9470080, rs737054, and rs9470079). We discovered that rs4713916A and rs6926133A were strongly linked to the comorbidity of severe DD susceptibility in MAUD, and the two SNP sites were in linkage disequilibrium (Figure 4).

The minor allele A of the rs4713916 polymorphism, which is present in the promoter region of the FKBP5 gene, is what causes the increased production of the FKBP5 protein. A meta-analysis study showed that the rs4713916 polymorphism was associated with mood disorders (56). The findings of another study showing that abuse and the rs4713916 polymorphism interact to impact the externalizing features of physiological anxiety were also supported by the findings in the present study (57). Interestingly, Russo et al. (58) found that FKBP5 rs4713916 may a useful predictor of clinical outcomes for pharmacogenomic intervention for chronic obstructive pulmonary disease (58). According to Du et al. (59) there was a significant difference in the frequency of the minor allele genotype of FKBP5 rs4713916 between the group of pediatric patients with primary nephrotic syndrome and the controls (*p* = 0.024) and between the group with steroid-dependent nephrotic syndrome and controls (*p* = 0.041) (*p* = 0.041) (59). In contrast, few investigations regarding FKBP5 rs6926133 in physiological disease have been described. However, rs6926133 has not been causally linked to the vulnerability-stress model of schizophrenia, according to Mihaljevic et al. (60).

## Conclusion

Our research here revealed that the FKBP5 risk alleles (rs4713916A and rs6926133A) are associated with the elevated comorbidity of severe DD in MAUD. Our findings shed light on the prediction of depression susceptibility in patients with MAUD and aid in the development of potential early warning biological indicators. Further investigation with large samples at multiple clinical facilities should be carried out to verify our conclusion. Among MA users and patients with MAUD, we should pay closer attention to the individuals with these two SNPs and provide them with individualized treatment.

## Data availability statement

The datasets presented in this study can be found in online repositories. The names of the repository/repositories and accession number(s) can be found in the article/Supplementary material.

## Ethics statement

The studies involving human participants were reviewed and approved by the Ethics Committee of the Second Xiangya Hospital of Central South University (No. 2017-064). The patients/participants provided their written informed consent to participate in this study. Written informed consent was obtained from the individual(s) for the publication of any potentially identifiable images or data included in this article.

## Author contributions

HL conceptualized and designed the research. TF and M-NL prepared the clinical samples and analyzed the data. X-YT, G-YL, and FL helped in preparing the analysis tools. XZ and WH conducted data interpretation. TF prepared the figures. HL wrote the manuscript. JL and NW provided the critical revision of the manuscript. All authors made substantial contributions to this study, critically reviewed the content, and approved the final version for publication.
