# Supplementary material for: The association of FKBP5 polymorphisms with the severity of depressive disorder in patients with methamphetamine use disorders
Source: Front Psychiatry. 2023 Mar 27;14:1147060. doi: 10.3389/fpsyt.2023.1147060 (PMC10083280; doi:10.3389/fpsyt.2023.1147060)
Supplement: Supplementary file 1 [file Table_1.docx]

**Supplementary Table 1.**

Primer information for gene SNP amplification.

| Gene SNP name | Primer sequence | Product (bp) |
| --- | --- | --- |
| rs4713916 | Forward: 5’-CTGGCTGGAATGTTGTATTT-3’ | 433 |
|  | Reverse: 5’-AAGTTGAGGCAGTAGAATGG-3’ |  |
| rs6926133 | Forward:5’-ATCTTTGTTCCCTCCATCTA -3’ | 810 |
|  | Reverse:5’-ATCCTCTTGGCTCTACCTAC-3’ |  |
| rs9470080 | Forward:5’-AAAAGAGGTGGCAGTTA-3’ | 714 |
|  | Reverse:5’-AGACAGGGTCCTACTTG-3’ |  |
| rs737054 | Forward:5’-ACTCAAATGATCCTCCCACT-3’ | 559 |
|  | Reverse:5’-CCACCCTTGTCTTGATGAACT-3’ |  |
| rs4713902 | Forward:5’-TTTGGTTCTCACCTTGCTAT-3’ | 323 |
|  | Reverse:5’-CTTGGAAAATTCAGTTTATG-3’ |  |
| rs737054 | Forward:5’-ACTCAAATGATCCTCCCACT-3’ | 559 |
|  | Reverse:5’-CCACCCTTGTCTTGATGAACT-3’ |  |
| rs9470079 | Forward:5’-CATTTGCTTAGCCTCTT-3’ | 805 |
|  | Reverse:5’-GATGCTTTCTTCCCTCA-3’ |  |

**Supplementary Table 2.**

Hardy-Weinberg Equilibrium analysis of genotype frequencies in the 6 FKBP5 SNPs

| SNP |  | Genotype | Observed | Expected | χ^2^ | P |
| --- | --- | --- | --- | --- | --- | --- |
| rs737054 | non-coding transcript exon variant | | |  | 0.575 | 0.448 |
|  |  | GG | 157 | 159 |  |  |
|  |  | AG | 110 | 105 |  |  |
|  |  | AA | 15 | 17 |  |  |
| rs6926133 | intron variant |  |  |  | 0.196 | 0.658 |
|  |  | CC | 168 | 169 |  |  |
|  |  | AC | 101 | 98 |  |  |
|  |  | AA | 13 | 14 |  |  |
| rs4713902 | intron variant |  |  |  | 0.443 | 0.506 |
|  |  | TT | 165 | 167 |  |  |
|  |  | CT | 104 | 100 |  |  |
|  |  | CC | 13 | 15 |  |  |
| rs9470080 | intron variant |  |  |  | 1.556 | 0.212 |
|  |  | CC | 120 | 125 |  |  |
|  |  | CT | 135 | 126 |  |  |
|  |  | TT | 27 | 32 |  |  |
| rs9470079 | intron variant |  |  |  | 1.774 | 0.183 |
|  |  | GG | 113 | 118 |  |  |
|  |  | AG | 139 | 129 |  |  |
|  |  | AA | 30 | 35 |  |  |
| rs4713916 | intron variant |  |  |  | 1.980 | 0.159 |
|  |  | GG | 162 | 166 |  |  |
|  |  | AG | 109 | 101 |  |  |
|  |  | AA | 11 | 15 |  |  |
